# Virtual colonoscopy, optical colonoscopy, or fecal occult blood testing for colorectal cancer screening: results of a pilot randomized controlled trial

**DOI:** 10.1186/s13063-015-0826-7

**Published:** 2015-07-09

**Authors:** John J. You, Yudong Liu, John Kirby, Parag Vora, Paul Moayyedi

**Affiliations:** Department of Medicine, McMaster University, 1280 Main Street West, Room HSC-2C8, Hamilton, Ontario L8S 4K1 Canada; Department of Clinical Epidemiology & Biostatistics, McMaster University, 1280 Main Street West, Room HSC-2C8, Hamilton, Ontario L8S 4K1 Canada; Faculty of Dentistry, Western University, Dental Sciences Building, Room 1003, London, Ontario N6A 5C1 Canada; Department of Radiology, Connolly Hospital Blanchardstown, Dublin, Ireland; Department of Radiology, McMaster University, 1280 Main Street West, Room 2S284, Hamilton, Ontario L8S 4K1 Canada

**Keywords:** Colonoscopy, Colorectal cancer, Computed tomography colonography, Fecal occult blood testing, pilot study, Prevention, Randomized controlled trial, Screening, Virtual colonoscopy

## Abstract

**Background:**

No head-to-head randomized controlled trials have demonstrated the superiority of one colorectal screening modality over another in reducing colorectal cancer mortality. We conducted a pilot randomized controlled trial of fecal occult blood testing (FOBT), optical colonoscopy (OC), and virtual colonoscopy (VC), to inform the planning of a larger evaluative trial.

**Methods:**

Eligible patients (aged 50 to 70) were recruited from five primary care practices in Hamilton, ON, Canada, between March 23, 2010 and August 11, 2010, and randomized 1:1:1 in a parallel design using an automated, centralized telephone service to either FOBT, OC, or VC. To reflect conventional practice, patients received no additional reminders to complete their allocated screening test beyond those received in usual practice. The primary outcome was completion of the assigned screening procedure. Results of the index test and any follow-up investigations were ascertained at 6 months. Participants, caregivers, and outcome assessors were not blinded to group assignment. The trial was stopped early due to lack of ongoing funding.

**Results:**

A total of 198 participants were enrolled, of whom 67 were allocated to FOBT, 66 to OC, and 65 to VC. The allocated screening procedure was completed by 43 (64 %) subjects allocated to FOBT (95 % confidence interval [CI], 52–75 %), 53 (80 %) subjects allocated to OC (95 % CI, 69–88 %), and 50 (77 %) subjects allocated to VC (95 % CI, 65–85 %); because the trial stopped early, we had insufficient statistical power to detect clinically relevant differences in completion rates. During 6 months follow-up, colorectal adenomas were detected in 0 (0 %) subjects allocated to FOBT, 12 (18 %) subjects allocated to OC, and 2 (3 %) subjects allocated to VC. One subject in the OC arm had histological evidence of high-grade dysplasia. No subjects were diagnosed with colorectal cancer.

**Conclusions:**

In this pilot randomized controlled trial of colorectal cancer screening in a primary care setting, 64–80 % of subjects completed their allocated screening test. These findings may be of value to investigators planning clinical trials to evaluate the effectiveness of colorectal cancer screening.

**Trial registration:**

ClinicalTrials.gov NCT00865527. https://clinicaltrials.gov/ct2/show/NCT00865527

## Background

Colorectal cancer is a leading cause of cancer death worldwide, killing an estimated 694,000 people each year [1]. There is evidence that certain colorectal cancer screening modalities may reduce the incidence of adenocarcinoma and that all approaches lead to the detection of earlier stage disease [2]. Current practice guidelines recommend a range of options for colorectal cancer screening, including fecal occult blood testing (FOBT), optical (endoscopic) colonoscopy (OC) and virtual colonoscopy (VC), also known as computed tomography colonography [3, 4]. However, these guidelines do not endorse a single best procedure for colorectal cancer screening, primarily because there are no head-to-head randomized controlled trials demonstrating that one screening test is more effective than another in reducing colorectal cancer mortality. We conducted a pilot randomized controlled trial to estimate the rates of completion of assigned screening procedures for patients allocated to FOBT, OC, or VC in order to inform the planning of a larger evaluative clinical trial.

## Methods

### Participants and setting

Patients age 50 to 70 years, identified from rosters of 5 participating family practices in the greater Hamilton region of Ontario, Canada, were eligible to participate in this study. Patients were excluded if they: were unable to give informed consent; had a history of colorectal cancer, adenomatous polyp, or inflammatory bowel disease; had undergone sigmoidoscopy, OC, or VC within the previous 3 years; had undergone FOBT screening in the previous year; or, if they had severe or terminal illness that would preclude any benefit from colorectal cancer screening. The McMaster University Faculty of Health Sciences/Hamilton Health Sciences Research Ethics Board approved this study (file number 09–147) and all participants gave written informed consent.

### Study procedures

Patients were first mailed a one page invitation letter on their family physician’s letterhead to participate in a study of colorectal cancer screening. The letter stated that colon cancer can be cured if detected early through screening and that patients who choose to participate in the study would be assigned at random to receive either FOBT, OC, or VC. Interested patients were invited to call a central booking office to schedule an appointment with the study nurse in order to learn more about the study. During the appointment, the study nurse enrolled eligible patients into the study. Patients who did not respond to the initial mailing were contacted once more either with a mailed reminder or a telephone follow-up call [5].

At the time of enrolment, we randomized patients in a 1:1:1 ratio using a parallel group design to colorectal screening with FOBT, OC, or VC. We used random variable block sizes and stratified by family physician practice and family history of colorectal cancer. Family history was obtained from an interview with the patient at the time of enrolment by the study nurse and was defined as a history of colorectal cancer or adenomatous polyps in a first-degree relative (parent, sibling, or child) at any age. We used an automated, centralized, telephone-based randomization service to maintain concealment of the computer generated allocation sequence. Patients allocated to FOBT were given the test kits along with educational materials and instructions for completion at the time of enrolment. Patients allocated to VC or OC were booked for these procedures using the conventional booking procedures for these investigations at our institution.

FOBT was performed by the patient at home using the guaiac-based FOBT kit recommended by the provincial Ontario Colon Cancer Check screening program (hema-screen, Immunostics Inc., Ocean, NJ, USA). Patients submitted the completed FOBT kit to a community laboratory and test results were sent to the patient’s family physician and the study methods center.

Patients randomized to OC took a bowel preparation regimen the day before the procedure, consisting of: four 5 mg tablets of bisacodyl, one sachet of sodium picosulfate 10 mg, magnesium oxide 3.5 g, and citric acid 12 g (Picosalax, Ferring Inc., Toronto, Ontario, Canada) dissolved in 150 mL of water, and drinking at least 1 L of clear fluid. Patients took a second sodium picosulfate sachet dissolved in 150 mL of water and drank at least 1 L of clear fluid at bedtime. During the procedure, patients received intravenous sedation with midazolam and fentanyl as needed, with typical dosages of 3 mg and 100 micrograms respectively, and received continuous monitoring of heart rate and oxygen saturation with a pulse oximeter. Decisions to biopsy suspicious lesions or perform polypectomy were left to the discretion of the endoscopist. OC examinations were performed by staff gastroenterologists at our academic health sciences centre who were fully licensed for independent practice by the regulatory body in our province.

Patients randomized to VC took a bowel preparation regimen (same regimen as the OC arm). Two hours before VC, patients received a small volume of oral contrast, which consisted of 20 mL of diatrizoate meglumine/diatrizoate sodium (Gastrografin, E-Z-EM Canada, Anjou, Quebec, Canada) diluted in 200 mL of water to tag any residual feces in the bowel [6, 7]. Once positioned in the computed tomography scanner, patients were given a bowel antispasmodic, 20 mg of hyoscine butylbromide (or, if contraindicated, 0.1 mg of glucagon) intravenously. A mechanical insufflator was used to insufflate carbon dioxide through a rectal tube to maintain a steady pressure of 20 to 23 mmHg. Single breath-hold computed tomographs were acquired without intravenous contrast with the patient in the supine and prone positions (total radiation dose 200 milliamperes-seconds). Images were reviewed by radiologist promptly to determine whether follow-up OC for polypectomy or biopsy was needed. According to accepted practice at the time of the study, polyps were only reported if greater than 6 mm in size.

Abnormalities found on any of the screening tests were communicated to the participant’s family physician, and decisions about the follow-up of abnormal test results were left to the discretion of the family physician. Research staff contacted patients by telephone once at 6 months after the study intervention to ascertain whether the patient had undergone the study intervention and to document whether crossover to another study arm had occurred. At 6 months follow-up, research staff also reviewed patients’ medical records (at Hamilton Health Sciences, where the screening OC and VC procedures were performed, and at the participating family physician offices) to confirm the results of the index screening test and to collect data about further testing that was done to follow-up on findings from the index screening test. Patients, care providers, and outcome assessors were not blinded to the study intervention.

### Outcomes

The primary outcome was completion of the initial colorectal screening intervention to which the patient was allocated. In other words, FOBT was considered complete if the patient returned the FOBT kit to the lab, regardless of whether they underwent follow-up OC if the FOBT test was positive. Similarly, VC was considered complete if the patient underwent the VC examination, regardless of whether they underwent follow-up OC based on VC findings. Secondary outcomes were: the proportion of patients who crossed over to another arm of the study; results of the index screening test; for patients allocated to VC, the proportion of patients who received same or next day OC to follow-up results found on the index VC test; and the proportion of patients in whom colorectal adenoma or invasive carcinoma was found at 6 months follow-up.

### Sample size calculation

Using a conservative assumption that 50 % of patients in a given arm would complete their allocated screening test (a proportion of 50 % would yield the widest 95 % confidence interval) we required 196 patients to be enrolled in each arm to estimate the proportion of patients who complete screening with a 95 % confidence interval of plus or minus 7 %. Therefore, we sought to enroll 200 patients in each arm. This sample size would also have permitted detection of an absolute difference of 13 % or more in the primary outcome between any 2 arms with a significance level of 0.05 and 80 % power. However, the trial was stopped early due to lack of ongoing funding.

### Statistical analysis

Baseline clinical characteristics, primary and secondary outcomes are presented using descriptive statistics (i.e., mean and standard deviation for continuous variables and proportions for categorical variables). We compared the proportion of patients who completed their allocated screening test (primary outcome) across the 3 arms using a chi-squared test. Statistical tests were 2-tailed using a significance level of 0.05. Analyses of the primary outcome were conducted according to the intention-to-treat principle.

## Results

### Study cohort

**Fig. 1:**
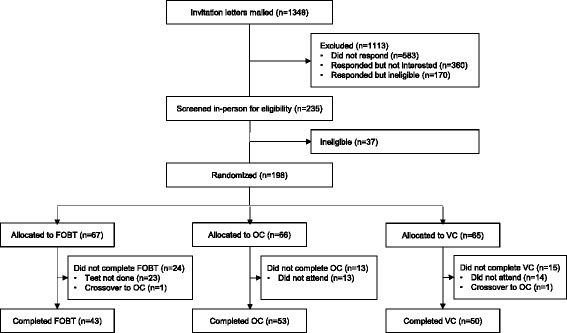
CONSORT study flow diagram

Participants were enrolled between March 23, 2010 and August 11, 2010 after which the trial was stopped early due to lack of funding. A total of 198 participants were enrolled, of whom 67 were allocated to FOBT, 66 to OC, and 65 to VC (Fig. 1). Baseline characteristics of participating patients were similar across the three groups (Table 1).

**Table 1:** Baseline characteristics of study participants

Characteristics	FOBT	Optical Colonoscopy	Virtual Colonoscopy	P*
	(*N* = 67)	(*N* = 66)	(*N* = 65)	
Age, years, mean (SD)	58.7 (5.4)	58.4 (5.6)	59 (5.6)	0.77
Female	31 (46)	25 (38)	38 (58)	0.06
Colorectal cancer in first degree relative(s)	9 (13)	9 (14)	8 (12)	0.97
Signs or symptoms				
Change in bowel habit	4 (6)	1 (2)	1 (2)	0.23
Blood in stool	3 (4)	0 (0)	1 (2)	0.18
Abdominal pain	0 (0)	0 (0)	0 (0)	1.00
>10 % weight loss in past 6 months	0 (0)	0 (0)	0 (0)	1.00
Unexplained anemia	0 (0)	0 (0)	0 (0)	1.00
Smoking history				
Never	36 (54)	34 (51)	29 (45)	0.63
Former	27 (40)	25 (38)	30 (46)	
Current	4 (6)	7 (11)	6 (9)	

Data are reported as number (percent) unless otherwise indicated. FOBT, fecal occult blood testing

*P value is for comparison of means or proportions across the 3 groups using one-way analysis of variance or chi-squared tests, respectively

### Completion of allocated colorectal cancer screening procedures

The allocated screening procedure was completed by 43 (64 %) subjects allocated to FOBT (95 % confidence interval [CI], 52 % to 75 %), 53 (80 %) subjects allocated to OC (95 % CI, 69 % to 88 %), and 50 (77 %) subjects allocated to VC (95 % CI, 65 % to 85 %) (*P* = 0.08) (Fig. 1). Non-adherence to the allocated screening procedure was due to non-completion of the test, with the exception of 2 subjects who crossed over to another arm (1 crossover from FOBT to OC arm; 1 crossover from VC to OC arm). No subjects were lost to follow-up for the primary outcome. No subjects experienced complications of virtual or optical colonoscopy (e.g., bowel perforation).

### Outcomes after colorectal cancer screening

Of the 43 subjects who underwent FOBT, 37 (86 %) tested negative, 4 (9 %) had indeterminate results, and 2 (5 %) tested positive; 1 of the latter 2 subjects went on to have a follow-up OC which did not reveal any polyps or mass lesions.

Of the 55 subjects who received OC as their index screening test (including 2 crossovers from other arms), 22 (40 %) subjects had at least one polyp, most of which were 5 mm or less in size, and 12 subjects had at least one colorectal adenoma, one of which had evidence of high grade dysplasia (Table 2).

**Table 2:** Findings on optical colonoscopy

	Patients with optical colonoscopy as index screening test^a^
	(*N* = 55)
Subjects with polyp(s)^b^	22 (40)
Largest polyp 5 mm or less	14
Largest polyp 6 mm to 9 mm	2
Largest polyp 10 mm or greater	4
Distribution of polyps by size, n†	
5 mm or less	33
6 mm to 9 mm	3
10 mm or greater	6
Distribution of polyps by histology, n	
Hyperplastic polyp	11
Tubular adenoma	8
Sessile serrated adenoma	3
Tubulovillous adenoma	1
Subjects with colorectal adenoma(s)	12 (22)^c^
Subjects with invasive carcinoma	0 (0)

Data are reported as number (percent) unless otherwise indicated

^a^includes 53 subjects originally allocated to OC and 2 subjects who crossed over to OC from other arms

^b^as visualized on OC; polyp size data missing for 2 subjects

^c^1 of these subjects had an adenoma with high grade dysplasia

Of the 50 subjects who received VC, 8 (16 %) had at least one polyp greater than 5 mm in size (since smaller polyps were not reported) and 5 (10 %) underwent follow-up OC to further investigate findings seen on VC (Table 3). Of the 5 subjects who underwent follow-up OC, colorectal adenomas were found in 2 subjects, none of which had evidence of high-grade dysplasia. After VC, follow-up OC was able be performed on the same or next day in 3 of the 5 subjects who were referred for follow-up OC.

**Table 3:** Findings on virtual colonoscopy

	Patients with virtual colonoscopy as index screening test
	(*N* = 50)
Subjects with polyp(s)^a^	8 (16)
Largest polyp 6 mm to 9 mm	7
Largest polyp 10 mm or greater	1
Distribution of polyps by size, n^a^	
6 mm to 9 mm	8
10 mm or greater	1
Patients who underwent follow-up optical colonoscopy	5 (10)
Distribution of polyps by histology, n	
Sessile serrated adenoma	3
Patients with colorectal adenoma(s)	2
Patients with invasive carcinoma	0

Data are reported as number (percent) unless otherwise indicated

^a^Only polyps greater than 5 mm in size were reported (see Methods)

In summary, at 6 months follow-up, colorectal adenoma(s) were detected in 0 (0 %) subjects allocated to FOBT, 12 (18 %) subjects allocated to OC, and 2 (3 %) subjects allocated to VC; one subject in the OC arm had histological evidence of high-grade dysplasia. None were diagnosed with colorectal cancer.

## Discussion

In this pilot, randomized controlled trial of colorectal cancer screening, adherence to assigned colorectal cancer screening was 64 %, 77 %, and 80 % for patients allocated to FOBT, VC, and OC, respectively. Although the trial was stopped early, the findings may be useful to investigators planning randomized controlled trials of colorectal cancer screening in a primary care setting.

Our findings that 20 % to 36 % of patients did not complete their assigned screening test has several implications for a future evaluative trial. If the goal of a future trial were to assess the effect of these colorectal cancer screening interventions under ideal conditions (i.e., efficacy), then our findings suggest that modification of our study procedures and a greater commitment of resources would be needed to increase adherence rates in a future clinical trial. In contrast, if the aim of a future trial were to evaluate the effectiveness (not efficacy) of colorectal cancer screening under conditions more reflective of usual practice, as was our intent, our findings provide useful estimates of anticipated adherence to FOBT, VC, and VC under typical conditions, since we did not provide any additional reminders for participants to complete their assigned screening procedures beyond usual booking or testing procedures. In particular, these estimates of test completion would be necessary to inform sample size calculations for a larger evaluative trial since non-completion of screening procedures would dilute any effect of the screening interventions on clinical outcomes such as colorectal cancer mortality.

Our observed adherence to FOBT of 64 % is similar to that observed in the original trials of FOBT screening from the 1980s which documented adherence of 60 % and 67 % [8, 9]. In our trial, adherence was lower in the FOBT arm compared to the OC and VC arms, but the difference was not statistically significant. Other recent randomized controlled trials have reported data about screening completion rates. In one trial, participation rates were similar regardless of screening modality (30.1 % for FOBT delivered by mail, 28.1 % for FOBT delivered by a general practitioner or screening facility, and 28.1 % for once-only sigmoidoscopy) [10]. In another clinical trial, a statistically significant difference in participation rates of 34.2 % for fecal immunochemical testing versus 24.6 % for optical colonoscopy was observed [11]. However, participation rates in these two trials are difficult to compare directly to ours since they randomized patients identified from population registries before obtaining informed consent for participation in the study, whereas we did not randomize patients to a screening intervention until after they gave consent to participate in the study. Our estimates of adherence are most applicable to patients who respond to invitations from their primary care physician to participate in colorectal cancer screening, agree to participate in a clinical trial, and then undergo allocated screening under usual conditions.

Our study has some limitations. First, we did not collect data from patients about the reasons why they did not complete their allocated screening test (e.g., lack of interest, failure of appointment booking procedures), which could have provided additional information to improve completion rates in a future trial. Second, our study was stopped early due to lack of funding. As a result, the estimates of adherence rates are more imprecise than we originally sought and, because of the reduced statistical power, it is not clear whether the lower adherence rate we observed for patients allocated to FOBT compared to the VC and OC arms is a true difference. Nonetheless, even though our estimates of adherence rates were not as precise as we had wanted (our original sample size would have provided 95 % confidence intervals of, at most, plus or minus 7 %), we were still able to achieve a precision of approximately plus or minus 10 % with our reduced sample size. Moreover, we believe that it is important to fully report data from clinical trials that are stopped early for insufficient recruitment (due to lack of funding or other reasons), since the failure to do so threatens the integrity of clinical research, and since investigators have an ethical responsibility to trial participants, to funders, and to the scientific community to disseminate the findings of their research [12]. Finally, the overall participation rate (i.e., number of patients enrolled out of the total number invited) in colorectal cancer screening in our study was low (198/1,348 = 15 %). This may have been a constraint of our randomized design in which participating patients could not choose which screening procedure they would get. It is possible that patients willing to be enrolled in a clinical trial are different from those who agree to undergo colorectal cancer screening after it is offered by their usual care provider in routine clinical practice.

## Conclusions

In this pilot randomized controlled trial of colorectal cancer screening reflective of usual testing conditions in a primary care setting, we found that 64 % to 80 % of subjects completed their allocated screening test. These findings may be of value to investigators planning clinical trials to evaluate the effectiveness of colorectal cancer screening in primary care settings.

## Abbreviations

CI: confidence interval
FOBT: fecal occult blood testing
OC: optical colonoscopy
VC: virtual colonoscopy
